# Supplementary material for: Expression Patterns of Three Important Hormone Genes and Respiratory Metabolism in Antheraea pernyi during Pupal Diapause under a Long Photoperiod
Source: Insects. 2021 Aug 4;12(8):699. doi: 10.3390/insects12080699 (PMC8396664; doi:10.3390/insects12080699)

Fig.S1

ApPTTH gene

1	ATGATATCAAGATCTATCGTTATATTACTAGCATGCTCGGGGGTGTTGATTATAATGGAAGCACTAATGCCTAGAACAATGGCAATGAAA
1	M I S R S I V I L L A C S G V L I I M E A L M P R T M A M K
91	AGTACTAGAAACATACATGAATTCATGATTGAAGATCAAAGGACACGCAAGAAACACAATTACATGTTTCAAAGAGATAGGAACAACGAT
31	S T R N I H E F M I E D Q R T R K K H N Y M F Q R D R N N D
81	ATTTTACGGAATAAAAATAATGGTCTGATGTATGATATGGAATCTTTAGAAATAGATTCAAGCCCTGAAGATTATTCAAACCTCGTGATG
61	I L R N K N N G L M Y D M E S L E I D S S P E D Y S N L V M
71	GATTATGCAAACATGAAAAAGAACGATGTCTTTTTATTGGATAATTCCATAGAAACTAGAACGCGAAAAAGAGGCAATATTAAGCGTGTA
91	D Y A N M K K N D V F L L D N S I E T R T R K R G N I K R
361	AGTATTAAAATGCATTATATCAGCAACTTAACCACAGCTTGGTCCAGTTAAGTGCCTGCGCGCACTACGATGCAGTAAGTTGCGGTGCGG
451	TTTTTATTCCGCAATAAATAGTACGAGCCTGGGGTGGTATTTCCAGATTAGTCTAAGACCCCGTTATTACCAGCCCTTCCTTATTTCATA
541	TTACTTTATCGATTTTACATGTTTTTAATGACTCTTGAGAAAACAATATTATTTTACAATTACTTTTTTTTGCAACGTACTCTAATTATT
631	ATATCTTCCATGACGATGCTTTATCAAAACTGAATCACATTTATTTTGTAAACTGTACAAACAATAATAGTAATATACAATAGTAAAACG
721	TGTACTGTCAGTAAAATACATTTAATGAGCAACCGAAAAATGTGCAGTGAAAGGTTGCAAAAGACCTAAAGGCAATCAACCCGATTTAAA
811	TCCGGTGGGGAAATTTACCAGCAGTGCGCGCCTTTCCATACAATTCTATATGTTACAAATTTTGCGTTAAAAATTCGTGTGCATAAAAAC
901	CGTAATACCGTATCGGAAACTAACCCTCGAGAGCTACATAAGTAGTAATTGGTAAATGTACGGGTTAACTATAATAGATAATATTTTATT
991	TCATTTTCAGCAAAACATTCCTGATCCGCCTTGCAGCTGTGAGTATACAGTAAGTATGTTCATATAGCAATTTAAAACAACTTAATCCTT
120	Q N I P D P P C S C E Y T
1081	TACAAAAAATTGAAGGTTTAAAATTATTTTAAATTAATAAATTTACAGAATGAAACTGTAGATTTCGGCGAGAATGCTTTCCCGAGACAT
133	N E T V D F G E N A F P R H
1171	GTCGAATCTAGAAATTGTAGTGAGTTACGACAATCATCGTGTCTCTTTCCGTACGTTTGCAAGGAAACCTTATATGATGTAAGTGATATT
147	V E S R N C S E L R Q S S C L F P Y V C K E T L Y D
1261	ATTTTTAGATAATCTAAATTATCGTTGTACATTAATTTATTCGAGTTTGACCAACTAGTAAAGAAATGTCAGCAGAACTATCTGTCGGTT
1351	TACATTATTCAATAGTTTAGGAAATATTTCACTCATAAGGCACTTTCTGCCTATTTCTGGATTGGATATGATTATGTAAAAGTGTAACAT
1441	GGGATCTGTTAGTAATTTGGAATAAAATTGGGTGGGTTATGTCCGACTCGCACGGACCAAAACCCCACGGTGTACGTCGTCCGCTATAGT
1531	TGGGGCGGCACGAGATCTCTATAGACTTCATCGTGCCGCCCCAGCGGTTGGCTACATCTACACCAACTTGTGTCAAGTAAACTAGAGAAG
1621	CGATTCCCCACTTTTTTTAACTCAGCAACGTACTAACATAATCTTTATAACATAATCACAAATTCGCGGCACCCTTACATGCAAATGCAA
1711	CGAATTTTACACAGTTTCCTGGACTTTTTTTTTTTTTCTCGAATTGACTATTGACCCGGCAGGGTCTTCACAGCTTTCCTGGACAGTGAT
1801	CAACAACTAATCAAATCAAGCCGCTGTCCTAACCACCGCAAATACAGTTTTGAGGGATTTCAAAGAAACAATAAGAGATTGTATACTTAT
1891	TGTTTGTGTTAACAGTTTAACTTGTAAACACGTGCAATCAATAAATTTCGAGAAACTATTGTTTTGTACATTCATTCGAAATGACTCATT
1981	GGCAGACAGTTCGTGTTACAGAACTCATCGAAACATTTCTTTAAGTAACTGAGGGCTAATACTGAAATTACTATTTGAAGTTATAGAGGG
2071	TGCGTATTTTAAGTAATAGAGATTTTAGGCTCTGAGGGGAAGAGAGATTATTTTTTGAAATTATAGAGGTTTTTACTACGTCACAAAACT
2161	CACGAACTTTACCTCTTTATAATATTAATATAGATATAACAAATATGTTATAATAGTATAACGTTTAATTTTCTGTTAGACTTCTACTGT
2251	TATATTATTTTTCAGTGTACAATTAACATTCAAGAAAACCCCTAATATGGACAATTGTATCATGTACTCATAATGATATTCATCTTTTTA
2341	GATAAGCGTTTTAAAACGAAGACAATCAACGACGCAACCTTCTGAAAAAGTACCAAACGAATTGAAGTTTAGGTGGATTGCTGAAAAATG
173	I S V L K R R Q S T T Q P S E K V P N E L K F R W I A E K W
2431	GCAGATCAGTGTTGGATGTGTATGCACCAGGGACTACCGAGATACTATTAATCAAGATTAA
203	Q I S V G C V C T R D Y R D T I N Q D *

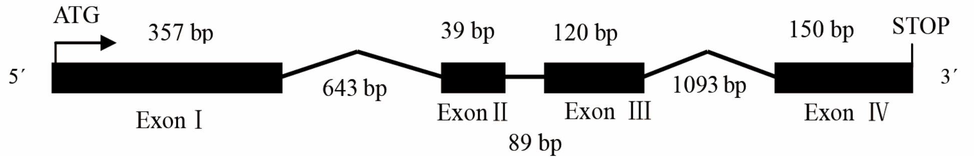

Fig.S2

ApETH gene

```
1     ATGAGTTTCAAATTGGCAACGGTCTTTGCAACATACTGCCTACTCTGCGTCCTAATCCAAATCGAAGCTTCGTTTATCAAACCTAACAAC
1      M  S  F  K  L  A  T  V  F  A  T  Y  C  L  L  C  V  L  I  Q  I  E  A  S  F  I  K  P  N  N
91    GTACCCAGAGTTGGACGGAGCAACGAGGCTTTGACTCCGTTTGATCAGGGGATGATGGGATACGTAATTAAAACCAATAAAAATATACCA
31     V  P  R  V  G  R  S  N  E  A  L  T  P  F  D  Q  G  M  M  G  Y  V  I  K  T  N  K  N  I  P
181   CGCATGGGAAGAAGAAATTATGATTCGGTAAGTTATAAAAAATTTTTTTTTTAATAACCGATATGTATAACTCGTTTGTAATTACTTGT
61     R  M  G  R  R  N  Y  D  S
71   ACATTGATTGTTTTCTTTCATCCATGGTTAGTCCAGTATAATAAGATTATATTAAAACTGACTTTTCTAGTTTGATTAGGTCCAACTTAC
361   ACTTTTACAAAAGGCCGGCAACGCACTTGTGACTCCCCCGGTGTTGCTAGTGTTTATGGACGGCGGTGATCACTTACCATCGGATGACCC
451   GTATGTTCGTTTGCCTCTTATAATATAAAAAAAATACTTCTGGAGTTTACGAACAATATAATATTTAATTTCATATGCCTTAATTTATTT
541   CCTGTATAATATTTTAATTTTACAATATAATATCATTTATTTTGTCTAAAGCACTTAGTTAAAAATAAGATCTGAAGATCATTAGTTTTA
631   TACAAAATTATTTTTAAACTAGCGGGCCGGGCGGAGGTTGTCCGCCCTTGATGATATTTTTTTAAAATCTAAAATTCAACCGCATGGCCA
721   CCTACCGGGTCCAATCTAAGCCATTCTCGGATCCACTCGAACACGCACACAAAATTTCATCAAAATCGGTCCAACTGTTTAGAAGGAGTT
811   CAGTGACATACACAAGTACAGAAGAAATGTATATATAAAGATGTACAAATATATAAATTAGTTAGCTGGTATCTAATAATTATTACTGGG
901   ATAGATACTTATTGAAATTTATTTTTTATTTATATTATTATTATTTAGGAAAACCGGTACAACATCCCAAAACTGTATCAATTTCCCGTC
70                                                     E  N  R  Y  N  I  P  K  L  Y  Q  F  P  V
991   GAAAATGCTGAATATTATGGAGGTACGTATACTATAAAGGAATATGATTTAACGTTTCTAAAAGTTATAACGTTACTAATATAAATTTTA
84     E  N  A  E  Y  Y  G
1081  ATATAATTTTAGATGAAGGACAAAGCAACACTCAAGAACAACTTGACACGTACTATGTTAAACAAATGGAAAATATGAAGAAATGA
91              D  E  G  Q  S  N  T  Q  E  Q  L  D  T  Y  Y  V  K  Q  M  E  N  M  K  K  *
```

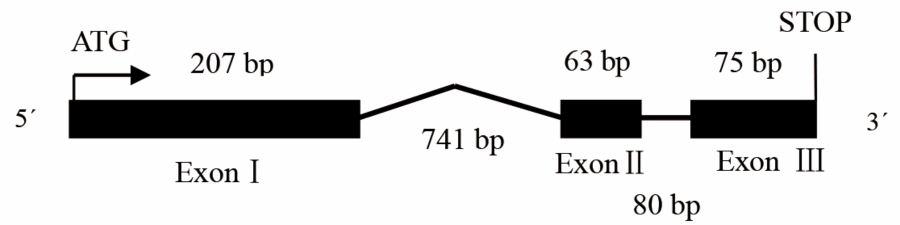

Fig.S3

ApEH gene

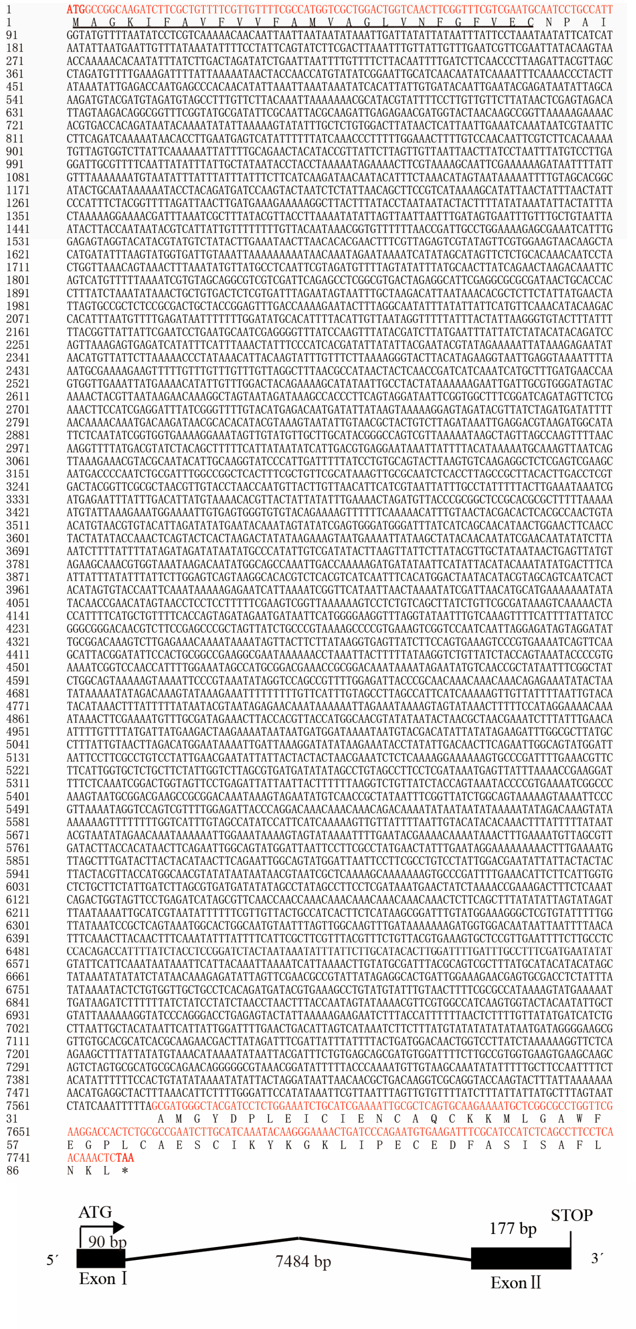

Supplement: Supplementary file 1 [file insects-12-00699-s001.zip › insects-1267073-supplementary.pdf]
